# Downburst-like experimental impinging jet measurements at the WindEEE Dome

**DOI:** 10.1038/s41597-022-01342-1

**Published:** 2022-05-27

**Authors:** Federico Canepa, Massimiliano Burlando, Djordje Romanic, Giovanni Solari, Horia Hangan

**Affiliations:** 1 Department of Civil, Chemical and Environmental Engineering (DICCA), Polytechnic School, University of Genoa, Via Montallegro 1, 16145 Genoa, Italy; 2 Wind Engineering, Energy and Environment (WindEEE) Research Institute, Western University, 2535 Advanced Avenue, London, Ontario N6M 0E2 Canada; 3 Department of Atmospheric and Oceanic Sciences, Faculty of Science, McGill University, Burnside Hall, 805 Sherbrook Street West, Montreal, Quebec H3A 0B9 Canada; 4 Faculty of Engineering and Applied Science, Ontario Tech University, 2000 Simcoe Street North, Oshawa, Ontario L1G 0C5 Canada

**Keywords:** Environmental impact, Atmospheric dynamics, Natural hazards, Civil engineering, Fluid dynamics

## Abstract

This paper describes the dataset of measurements collected and published in the context of the comprehensive experimental campaign on downburst-like outflows that was performed at the WindEEE Dome at Western University, Canada. Downbursts are strong downdrafts of air that originate from thunderstorm clouds and create vigorous radial outflows upon hitting the ground. Downbursts are here simulated as transient phenomena produced by large-scale impinging jet. Two jet velocities were adopted in the experiments. The three-component velocity measurements were recorded using 7 Cobra probes mounted on a vertical stiff mast and displaced at 10 radial positions in respect to the downdraft centerline. For every radial position, each experiment with the same initial condition was repeated 20 times to inspect the deterministic features of the signal. Overall, the total of 2800 tests (2 jet velocities × 20 repetitions × 10 radial positions × 7 heights) represent one of the largest experimental campaigns on downburst winds carried out in a wind tunnel facility thus far.

## Abstract (summary)

**Table:** 

Measurement(s)	atmospheric wind speed
Technology Type(s)	Cobra Probe
Factor Type(s)	wind velocity
Sample Characteristic - Organism	wind flow
Sample Characteristic - Environment	wind tunnel

## Background & Summary

**Fig. 1:**
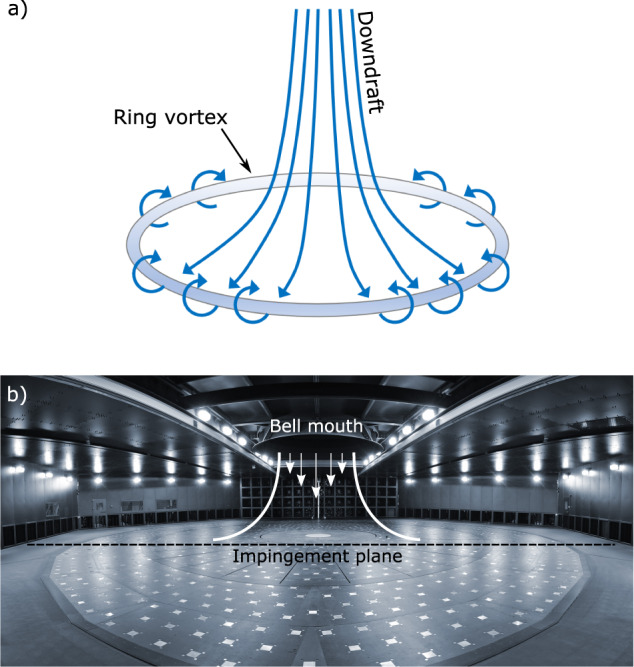
(**a**) Three-dimensional schematic of downburst (after Fujita^64^); (**b**) Testing chamber of the WindEEE Dome with schematic of the downburst-like IJ.

Downbursts are strong buoyancy-driven downdrafts of cold air that descend from thunderstorm clouds. Once the downdraft hits the surface, the velocities in the radially advancing outflow can exceed 75 m s^–1^ ^1^ in the first approximately 100–150 m from the surface, where the maximum flow intensities occur and give rise to the well-known nose-like shape vertical profile of velocity^2–4^. The outflow is led by the primary vortex (PV), which is a vortex ring that forms below the thunderstorm parent cloud because of the instability between the dense cold column of air descending to the ground and the calm surrounding environment. Fig. 1a represents a schematic of the downburst spatial evolution. The high wind speeds near the ground can pose a serious hazard to structure, aircraft, and the environment. The increasing intensity and frequency of occurrence of thunderstorms that seems to be linked to climate changes^5–7^ requires reassessment of thunderstorm wind impacts on the built environment^8^. On average, downbursts last for approximately 10 min^3,9^ and their spatial extent is not larger than several kilometers in diameter^3^. This makes downbursts small-scale, highly three dimensional (3D) and unsteady wind flows. Consequently, real measurements of downburst winds in nature are very challenging and still rare compared to larger-scale wind flows associated with atmospheric fronts and low-pressure systems. This lack of data and the complexity of downbursts often make it impossible to reconstruct the overall spatial structure of the downburst outflow by only relying on the standard anemometric records.

In parallel with the field measurements, downbursts have also been investigated computationally^10–15^. However, numerical models often lack the proper representation of turbulence and instabilities, mainly due to coarse spatial and time resolutions used in the discretization process of the governing equations. For these reasons, the physical modelling of downburst winds in experimental facilities has played increasingly more important role throughout the last decades. Two experimental approaches to create downburst-like flows in wind simulators are buoyancy-driven (gravity) currents^16–20^ and momentum-driven impinging jets (IJs)^21–25^. The velocity scale of gravity current experiments reported in the literature is often too small to facilitate reliable measurements of the kinematic properties of the near-ground wind field (i.e., a few millimeters above the floor of the testing chamber), which, however, is the layer of interest in a study of flow-structure interaction. Nevertheless, the facilities that are capable of creating downburst-like IJs^23,25,26^ are still few and sometimes limited by small scales or, for instance, continuous impingement of the jet that creates a 3D, but steady-state outflow near the surface. Currently, the largest geometric scales of experimentally produced IJ downbursts are achieved in the Wind Engineering, Energy and Environment (WindEEE) Dome^27^ at Western University (Canada). The reported geometric scales in their experiments were approximately 1:200 and larger^28,29^.

In this paper, a large database of measurements recorded during a recent experimental campaign in the WindEEE Dome is presented in terms of methodology, instrumentation, and specification of the spatiotemporal grid of measurement points. The related dataset is published online at the PANGAEA repository^30^. The large number of performed tests on the kinematics of downburst-like IJs enables the detailed study of the spatiotemporal evolution of the downburst outflow over the measuring instruments. The thorough dataset of the three-component wind speed that we present here has been carefully quality checked and analyzed in terms of both mean and turbulent components of the downburst outflow and can be further reused by other researchers. For example, we expect that the published dataset will be particularly useful for those researchers interested in validating and calibrating their numerical simulations of downburst winds, as well as for the comparison between IJ and buoyancy-driven outflows. In the following of this paper, our experimental data are validated against a real downburst in order to estimate the accuracy and applicability of the experimental method to replicate these types of wind events. Further comparisons with full-scale events can be assessed by other users of this dataset. We expect that the published data will be a valuable resource for those interested in atmospheric physics, meteorology, fluid dynamics, natural disasters modelling as well as the insurance industry and their assessment of losses arising from thunderstorm winds. Lastly, downbursts have been a focal point of wind engineering research and practice over the last few decades and there is an on-going effort towards the codification of downburst winds in building codes and wind loading recommendations. Historically, wind tunnel measurements have been the major resource for codification of atmospheric boundary layer winds^31^ and we expect that the provided dataset will be of similar importance in the codification of thunderstorm downbursts.

## Methods

### Facility

All downburst-like IJ experiments performed in this research were carried out in the WindEEE Dome at Western University, Canada^32^. The capacity of the WindEEE Dome to replicate atmospheric boundary layer, shear, tornadic, and downburst wind flows was presented in Hangan *et al*.^27^. In short, the WindEEE Dome is a hexagonal chamber of 25 m in diameter (Fig. 1b) surrounded by an outer return chamber of 40 m in diameter. The 25 m diameter testing chamber has 100 fans on the peripheral walls, out of which 60 are mounted on one of the six walls in a matrix of 4 rows and 15 columns and the rest of 40 fans are distributed at the base of the other five peripheral walls. An upper plenum with six larger fans is situated above the testing chamber. The test chamber and the upper plenum are connected through a bell mouth situated at the ceiling level of the test chamber (Fig. 1b). Fig. 1b is a photograph of the WindEEE Dome testing chamber.

### Downburst-like wind generation and flow intensities

**Fig. 2:**
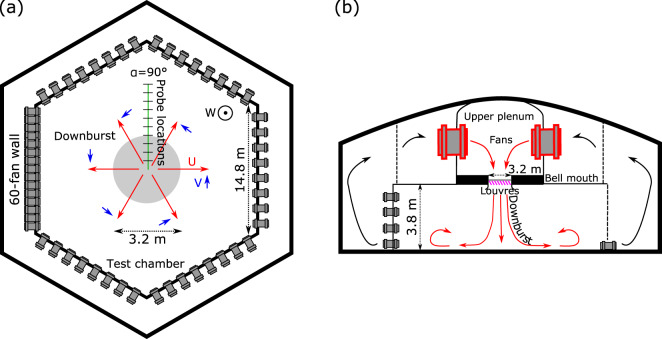
(**a**) Top and (**b**) side views of the WindEEE Dome downburst mode. Also, (**a**) shows the positive direction of velocity components (*U*, *V*, *W*).

Downburst-like flows in the WindEEE Dome are produced as schematically depicted in Fig. 2. Here, downbursts are simulated as pulsed IJs through the rapid opening and closing of mechanical louvres installed at the bell mouth level resulting in an IJ that runs through the bell mouth (maximum diameter *D* = 4.5 m) before impinging on the floor of the testing chamber. The process of creating a transient IJ has two steps. First, the upper chamber is pressurized using the upper fans and by closing the louvers on the bell mouth. Second, once the pressure reaches the desired pressure of approximately 3.4 hPa above the pressure value in the testing chamber^33^, the louvers of the bell mouth are opened, and the air is released into the testing chamber. The diameter of the bell mouth can vary from the maximum value of 4.5 m to the minimum of 1.2 m. A diameter of *D* = 3.2 m was used in this study. The corresponding *H*/*D* ratio was 1.17, where *H* = 3.75 m is the ceiling height of the testing chamber. Previous studies have demonstrated that the confinement effects in IJs with *H*/*D* >1 are negligible and thus the primary vortex at the leading edge of downburst outflow can fully develop^24,28,34^. This kinematics is consistent with the formation of the primary vortex in actual downbursts. The present experiments investigated two different intensities of IJs that were determined using the centerline jet velocities, *W*_*jet*_, at the exit of the bell mouth. The corresponding velocities were 8.9 and 16.4 m s^–1^ ^33^. Hence, the two cases are hereafter referred to as DB8.9 and DB16.4, respectively. The inflow characteristic velocities associated with the experiments are summarized in Table 1. The dynamical characteristics of various IJs, included the velocity and turbulence distribution, at the exit of the bell mouth were recently investigated by Romanic *et al*.^33^, Romanic and Hangan^35^ and Junayed *et al*.^28^.

**Table 1:** Experiment setup summary.

Case name	*D* [m]	*W*_jet_ [m s^−1^]	*r* [m]	*r/D*	*z* [m]	*z/z*_max_	Reps
DB8.9	3.2	8.9	0.64–6.4*	0.2–2.0**	0.04, 0.10, 0.15, 0.20, 0.27, 0.42, 0.50	0.4, 1.0, 1.5, 2.0, 2.7, 4.2, 5.0	20
DB16.4	3.2	16.4	0.64–6.4*	0.2–2.0**	0.04, 0.10, 0.15, 0.20, 0.27, 0.42, 0.50	0.4, 1.0, 1.5, 2.0, 2.7, 4.2, 5.0	20

*The radial increment was ∆*r* = 0.64 m. ** The radial increment was ∆*r/D* = 0.2. zmax = 0.1 m.

### Velocity measurements setup

**Fig. 3:**
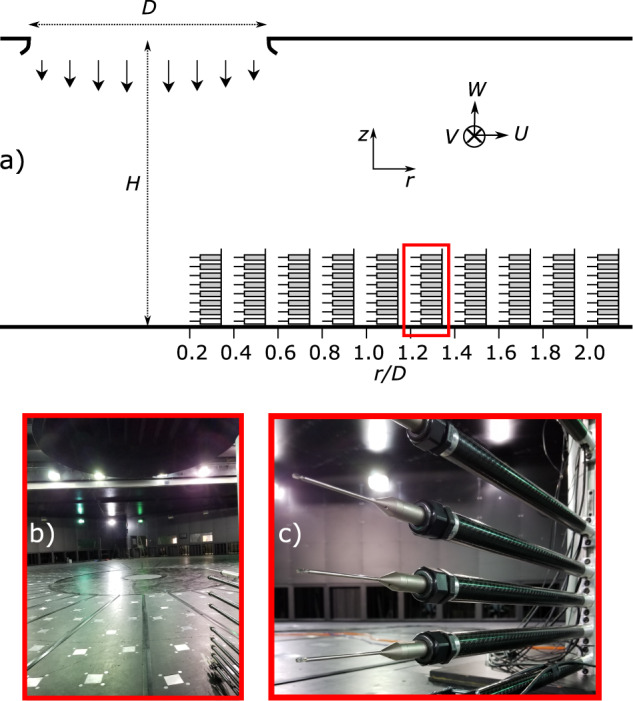
(**a**) Downburst measurements experiment setup and the coordinate system (*U*, *V*, *W*). The bell mouth diameter and chamber height are *D* = 3.2 m, *H* = 3.75 m, respectively. The Cobra probe heights are 0.04, 0.10, 0.15, 0.20, 0.27, 0.42, and 0.50 m above floor. (**b**) Photo of Cobra probe rack (the red rectangle in (a)) facing the direction of downburst touchdown (i.e. *r/D* = 0) in the center of turntable. (**c**) A closer look at Cobra probes and the mounting booms.

The experiments setup is depicted in Fig. 3. The flow was measured at different *r/D* positions using Cobra probes mounted on a heavy and stiff mast that prevented vibrations of the probes in the flow. Here, *r* is the radial distance from the jet centerline and *D* = 3.2 m is the jet diameter (see previous section). As the flow is expected to be axis-symmetric, measurements were taken only at the azimuthal angle *α* = 90° (see the rack position indicated in Fig. 2a). The ten radial positions, *r/D*, of the mast were in the range 0.2–2.0 with an increment of 0.2. The heights (*z*) of the probes on the mast were 0.04, 0.10, 0.15, 0.20, 0.27, 0.42 and 0.50 m above the testing chamber floor. Table 1 reports the probe measurement positions. The horizontal and vertical distances were normalized to the position of maximum slowly-varying radial velocity, $\hat{\bar{U}}$, in the outflow. In both IJ experiments, this characteristic velocity was at *r*_max_ = *D* = 3.2 m and *z*_max_ = 0.1 m. For every *r/D* position, each IJ experiment with the same initial conditions was repeated 20 times to inspect the repeatability of the tests and to build more statistical significance of the results; including the analysis of their variability. Therefore, a total of 2 jet velocities × 20 repetitions × 10 radial locations × 7 measuring probes resulted in 2800 velocity records. Table 1 summarizes the experiments setup.

Cobra probes are multi-hole pressure probes designed to resolve three components of velocity in real time. The velocity components are conventionally named (*U*, *V*, *W*) for the component along, transversal and vertical to the probe axis (see Fig. 3a), respectively. In this experimental setup (Fig. 2a), *U* is also the radial outflow of the downburst. The probes provide reliable measurements for flows approaching the probe within a cone of ±45°. The reported manufacturer’s accuracy of the probes is ±0.5 m s^–1^ in velocity measurements and ±1° yaw and pitch angles up to approximately 30% of turbulence intensity. The sampling frequency of all velocity measurements was *f*_*s*_ = 2500 Hz, which is a sufficiently high sampling frequency in turbulent flows. All velocity magnitudes below 1 m s^−1^ were removed and converted to NaN (Not a Number) from the published database due to the poor accuracy of Cobra probes for velocities below this threshold. In addition to NaNs, some velocity values were reported as a null in the instrument readings even in the cases when the velocities around the null value were generally above 1 m s^−1^. These null readings were likely caused by the sudden change of wind direction in turbulent flows and the incoming flow being outside the probe’s spatial cone of measurement. These values are flagged as NULL values in the database. During the testing, the air temperature and air density in the chamber were approximately 296 K and 1.148 kg m^–3^, respectively. With the upper chamber being pressurized, the louvers of the bell mouth were suddenly opened and 3–5 s later suddenly closed to create transient downburst-like IJs. The closing procedure was relying on manually pushing a button in the control room and hence the uncertainty of approximately 2 s. This uncertainty was an additional motivation for conducting 20 repetitions of each test. The measurement interval in each repetition was 20 s long, but the velocity records saved in the final database uploaded to the PANGAEA repository are 10 s long (25,000 samples). The uploaded velocity records are the segments of the original time series containing only the downburst signal that was trimmed following the data handling procedures described in the following section.

### Data synchronization

Hereafter, our analyses mainly focus on the radial velocity component, *U*, that represents the dominant velocity in a downburst-like outflow.

**Fig. 4:**
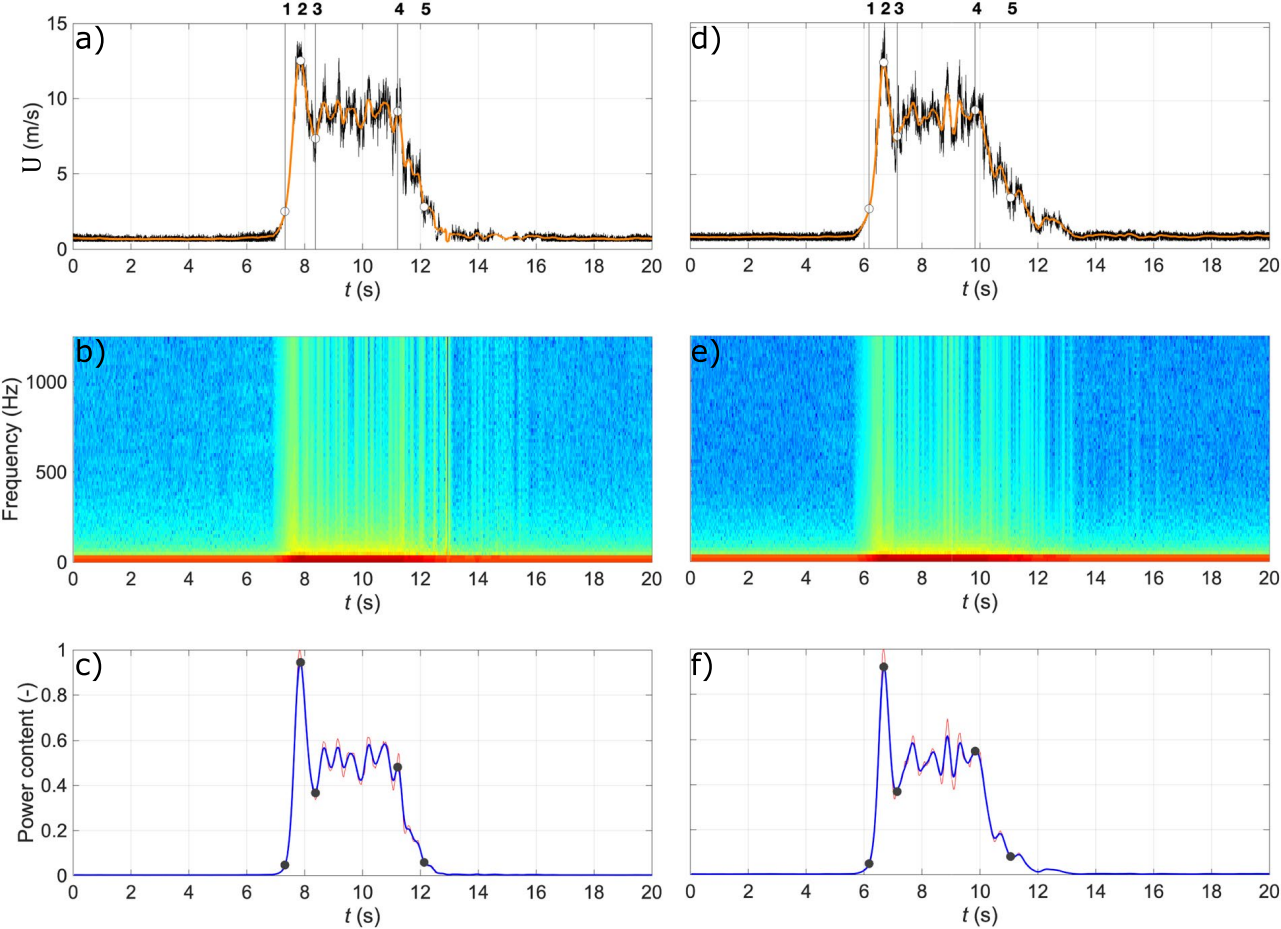
Downburst test repetitions #9 and #20 at the position (*r*/*r*_max_ = 1.0, *z*/*z*_max_ = 1.0) and for the case DB8.9: (**a**,**d**) time series of radial wind speed (black line) and its moving average (orange line); (**b**,**e**) periodogram of power spectral density as 10log_10_ (*P*_*k*_); and (**c,f**) normalized power content.

Data synchronization from 20 repetitions was performed using a time-dependent spectral analysis of the times series. This analysis was performed in MATLAB^®^ (see ‘1_alignSignals.m’^36^). To depict this methodology, Fig. 4a,d shows time-series (black line) of the radial wind speed and the corresponding moving average (orange line) evaluated over a time window of 500 samples (i.e., 0.2 s) for the repetitions #9 and #20 recorded at the position (*r*/*r*_max_ = 1.0, *z*/*z*_max_ = 1.0). Fig. 4b,e are the periodogram power spectral density (PSD) of the velocity records calculated using 27 points in the segmented windows. The goal here is to divide a velocity record into shorter portions and then independently compute the fast Fourier transform on each of the segments. The spectra (*S*_*k*_) at the frequency *k* is computed using the short-time discrete fast Fourier transform in the form of modified periodograms as follows:

1 $${S}_{k}=\mathop{\sum }\limits_{n=0}^{N-1}{h}_{n}{U}_{n}{e}^{\frac{-i2\pi k}{N}}$$

where *N* is the number of velocity readings in each spectral window, *U*_*n*_ is the wind speed at the *n*-th reading in the window, *k ∈* (0, 1, 2, …, *N*–1), *h*_*n*_ is the periodogram modifier in the form of Hamming window and *I* = √(−1). This study uses a 50% overlap between adjacent windows to reduce the effects of windowing near the frame edges. The power spectral density (*P*_*k*_) is obtained as:

2 $${P}_{k}=\frac{1}{{f}_{s}}\frac{2{\left|{S}_{k}\right|}^{2}}{H}$$

where $H=\mathop{\sum }\limits_{n=0}^{N-1}{h}_{n}^{2}$ is the window normalization constant and *f*_*s*_ = 2500 Hz is the sampling frequency. Lastly, the normalized power content (*P*_*t*_) in Fig. 4c,f is calculated as:

3 $${{\mathcal{P}}}_{t}=\frac{1}{\widehat{{\mathcal{P}}}}\mathop{\sum }\limits_{k=0}^{\frac{{f}_{s}}{2}+1}{P}_{k,t}$$

where $\widehat{{\mathcal{P}}}=\mathop{\max }\limits_{t}\left({{\mathcal{P}}}_{t}\right)$ and *t* is the time. The proposed methodology for aligning the velocity signals uses the moving average of *P*_*t*_ calculated every 10 samples (which correspond approximately to 0.5 s), hereafter ${\widetilde{{\mathcal{P}}}}_{t}$, with the threshold value of ${\widetilde{{\mathcal{P}}}}_{t}$ = 0.1 as the starting pivot point for all 20 experiment repetitions. Then, the first local maximum after this point corresponds to the primary peak. Since the first peak occurs systematically in all analyzed velocity records, this dominant feature of the flow was used to shift the signals in time domain and synchronize them. Therefore, the time instant at which the power content (Fig. 4c,f) is at the maximum was used as a reference time for the alignment of velocity records from multiple repetitions of the same experiment.

The described signal alignment was computed only for the probe located at *z* = 0.1 m on the rake. The specific probe was chosen based on the maximum velocity which is indeed recorded at this height. Hence, this alignment is applied to all other probes on the rake that were sampled synchronously in order to maintain the time-evolution pattern of the flow along the vertical profile. For example, this alignment procedure allows the proper assessment of the cross-correlation of the turbulence components among the measurement heights.

## Data Records

The presented database of downburst-like measurements in the WindEEE Dome is made available through the data publisher for Earth & Environmental Science PANGAEA^30^. The database is subdivided into two ASCII text files associated with two different jet velocities, *W*_*jet*_, reported above. The database reports 10-s time series of the three velocity components of the 20 experiment repetitions, after synchronization (see ‘Data Synchronization’ section), measured at 7 heights and 10 radial positions.

### Velocity signal segments

In repetition #9 (Fig. 4a), the bell mouth was opened about 7 s after the initiation of the sampling procedure. After the bell mouth louvers opened, the vortex ring (Fig. 1a) produced by the downdraft in the form of a primary vortex (PV) hit the ground, expanded radially, and reached the Cobra probe (time instance 1 in Fig. 4a). The measurements continued until the downdraft was terminated approximately 5–6 s from the bell mouth opening. The passage of the PV by the Cobra probe is marked by the first peak in the time series which also corresponds to the time instant “2” in Fig. 4a. The end of the primary peak is the first local minimum after the peak itself (time instant 3). Afterwards, the steady-state velocity segment starts and ends with the last local maximum in the record (time instant 4). Finally, the end of downburst record is when ${\widetilde{{\mathcal{P}}}}_{t}$ crosses the threshold value for the last time (time instant 5).

The two records reported in Fig. 4, as well as all 20 repetitions, are characterized by three different segments that correspond to three distinct periods in the downburst outflows observed in typical real events^37–39^. The first segment is associated with the passage of the primary vortex (PV) by the probe and accordingly this portion of the record is called the “PV” segment. In Fig. 4, the PV segment contains the ramp-up part of the signal (time instances 1 to 2), the first peak (time instant 2 is always the global maximum as well), and the velocity slowdown after the peak (time instances 2 to 3). After the PV segment, a steady state outflow—which is called the “plateau” segment in this study (time instances 3 to 4 in Fig. 4)—is characterized by a mean that is fairly constant over time with random fluctuations superimposed. In this segment, the signal fluctuations have typically different phases in different repetitions. The third segment in experimental velocity records is called the “dissipation” segment and it represents the interval when the downburst dissipates. During this segment, all velocity profiles decrease gradually to a near-zero value (time instances 4 to 5 in Fig. 4). The duration of each downburst record also depends on the radial distance from the IJ touchdown. Wilson *et al*.^40^ and Hjelmfelt^3^ analyzed 38 and 27 full-scale downburst events, respectively, and reported average values of downdraft radius, maximum outflow radial velocity and downburst duration of 900 m, 12 m s^−1^ and 16 min, respectively. More recently, in the context of some simulations carried out with Xhelaj *et al*.^41^ downburst analytical model, 10 downburst events were analyzed from the anemometric recordings in the main ports of the Northern Tyrrhenian Sea^42,43^. The same average quantities mentioned above were found equal to 1040 m, 20 m s^−1^ and 26 min, respectively. By considering the IJ radius used in this study, i.e., *R*_exp_ = *D*/2 = 1.6 m, and the reported values of full-scale downdraft radii, i.e. *R*_FS_ ≅ 1000 m, we obtain a length scale of Λ_*l*_ = *R*_FS_/*R*_exp_ = 625. The maximum radial wind speeds in our experimental campaign were approximately 13 m s^−1^ and 26 m s^−1^ for the cases DB8.9 and DB16.4, respectively. It is thus reasonable to assume a velocity scale Λ*v* ≅ 1. The time scale is therefore equal to the length scale, Λ_*t*_ = 625. The downburst-like part of the velocity records lasted 3–5 s (Fig. 4), which when scaled up is comparable with the field observations reported in the above studies. Any longer duration of the experimentally produced downburst-like jets affects only the steady-state part of the signal, i.e., the plateau segment, and therefore has no influence on the transient parts of the records (the PV and dissipation segments).

### Velocity signals reported in the database

**Fig. 5:**
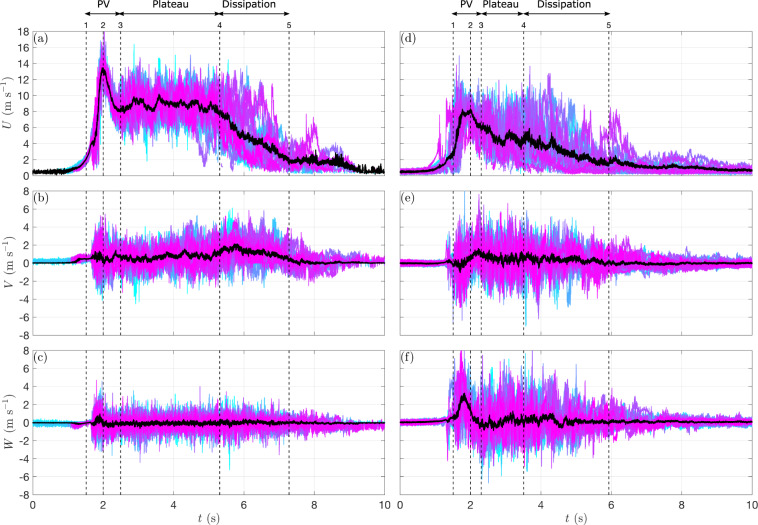
(**a**–**c**) 20 repetitions showing the radial, transversal and vertical wind speed components (*U*, *V*, *W*) (colored lines) measured at the position (*r*/*r*_max_ = 1.2, *z*/*z*_max_ = 0.4) for the case DB8.9 and their ensemble mean (black line) calculated according to Eq. (4); (**d**–**f**) Same as (**a**–**c**) but for the position (*r*/*r*_max_ = 1.8, *z*/*z*_max_ = 2.7).

For every investigated position in the flow (*r/r*_max_, *z*/*z*_max_), the corresponding 20 repetitions were aligned as described in the previous section (MATLAB^®^ script ‘1_alignSignals.m’^36^), then saved in a matrix format (MATLAB^®^ script ‘2_saveData.m’^36^) and finally used to create the final database of velocity measurements (MATLAB^®^ script ‘3_datasetWriting.m’^36^). Fig. 5 show the radial, transversal and vertical velocity component (*U,V,W*) of all 20 synchronized signals recorded at the positions (*r*/*r*_max_ = 1.2, *z*/*z*_max_ = 0.4) and (*r*/*r*_max_ = 1.8, *z*/*z*_max_ = 2.7), respectively. Each velocity record was trimmed to have an overall length of 10 s with the starting point exactly 2 s (5,000 samples) before the primary peak. This 10-s time frame of the signals was ensured to be suitable to include all the relevant part of the downburst time series, i.e. calm environment before the IJ release, downburst occurrence and return to calm environment after downburst dissipation. The black lines in Fig. 5 represent an ensemble average of all repetitions, calculated as follows:

4 $$\left\langle U(t)\right\rangle =\frac{1}{M}\mathop{\sum }\limits_{i=1}^{M}{U}^{\left(i\right)}(t)$$

where *M* = 20 is the total number of repetitions, *t* is the time, and *U*^(*i*)^(*t*) is the *i*-th measured time series. Each set of 20 aligned records, as well as their ensemble mean, show the PV, plateau and dissipation segments. The intensity and duration of each of the three segments vary depending on the (*r/r*_max_, *z*/*z*_max_) position in the outflow (Fig. 5). Therefore, Fig. 5 depicts the applicability of the proposed alignment methodology. In Fig. 5, Eq. (4) was also applied to the transversal and vertical wind component.

## Technical Validation

The technical validation of the experimental campaign presented in this article can be assessed through three levels: (1) Cobra probe instrumentation; (2) wind tunnel tests; and (3) specifically the WindEEE Dome laboratory.

### Measurement instruments

Cobra probes are robust, flexible and easy-to-mount velocity measuring devices widely used in wind tunnel applications. Cobra probes are able to resolve unsteady velocity components because their pressure transducers are located in close proximity to their pressure sensing ports. Guo and Wood^44^ compared Cobra probes against hot-wire anemometers and demonstrated their capability to measure mean and fluctuating components of the velocity field in highly turbulent environments (turbulence intensities up to 35%). Some advantages of Cobra probes over hot-wires are their robustness, temperature insensitivity, ease of use, ability to withstand moderate knocks and contaminated flow^28^. Validation of Cobra probe measurements is also found in Chen *et al*.^45^, where probe measurements were compared with established data for fully developed pipe flow and good agreement was found. Mallipudi *et al*.^46^ determined the unsteady wake characteristics of rotating objects by employing Cobra probes. The wind tunnel calibration of Cobra probes for speed, direction and frequency returned values within the manufacturer’s quoted accuracies. A very large number of wind engineering experimental studies in the literature is based on the use of Cobra probes, including those with applications to non-stationary flows, including downburst winds^25,28,35,47–52^.

### Wind tunnel experimentation

Despite the scientific evolution of wind engineering makes available nowadays powerful analytical and numerical tools to solve a wide range of problems, there are still many situations in which such criteria are inadequate or with limited reliability. Full-scale measurements are often impractical due to exaggerated costs, organizational challenges, and long durations. Furthermore, they are often incompatible with the design requirements. With regard to downburst winds, only the availability of records from instruments such as LiDARs and multiple meteorological Doppler radars has allowed to achieve a more refined spatial resolution of the measurements at the very local scale close to the ground^37,42,53–56^. Numerical models are often limited by coarse resolution close to the ground and lack of suitable methods to properly represent turbulence. Wind tunnel tests, on the other hand, are efficient, quick and financially affordable operational tools. Wind tunnel tests are divided in two main subcategories: the first includes tests in the mechanical and aeronautical field to assess the aerodynamics of vehicles and aircrafts; the second category includes tests in the civil and environmental area to simulate and study various flow fields, transport and circulation of passive substances, and the investigation of wind actions and effects on the built environment. Wind tunnel tests are a well-established method worldwide and a reference tool in the cases when the design solutions do not fall within the standards provided by the building codes (see, for instance, ASCE^57^ and Eurocode^58^).

The last twenty years have seen a rapid rise of numerous ad-hoc laboratories created to reproduce non-stationary wind flows and phenomena, such as thunderstorm winds and tornadoes. The WindEEE Dome is, to date, the largest scale laboratory worldwide capable of physically simulating these kinds of phenomena.

### WindEEE Dome laboratory

Established in 2011, the Wind Engineering, Energy and Environment (WindEEE) Dome is the world first three-dimensional testing chamber.

Contrary to the usual small scales of previous experimental studies in the literature on non-stationary winds^23,25,59,60^, WindEEE Dome can reproduce downburst and tornado winds at the largest scales produced thus far in a wind simulator. Here, the geometric scale, defined as the ratio between the size of full-scale and the physically modelled quantities, is reported to be 200 or less, while the velocity scale is usually determined in the range 1.5 to 4^28,29,61,62^.

The Reynolds number, ${\rm{Re}}=\frac{{W}_{{\rm{jet}}}\times D}{\nu }$, of the downburst-like IJs produced at the WindEEE Dome is ~10^6^, which is significantly larger than the values reported in other wind simulators. The same order of magnitude of *Re* is found by expressing it as function of the maximum radial velocity $\widehat{\bar{U}}$ and its height of occurrence *z*_max_.

**Fig. 6:**
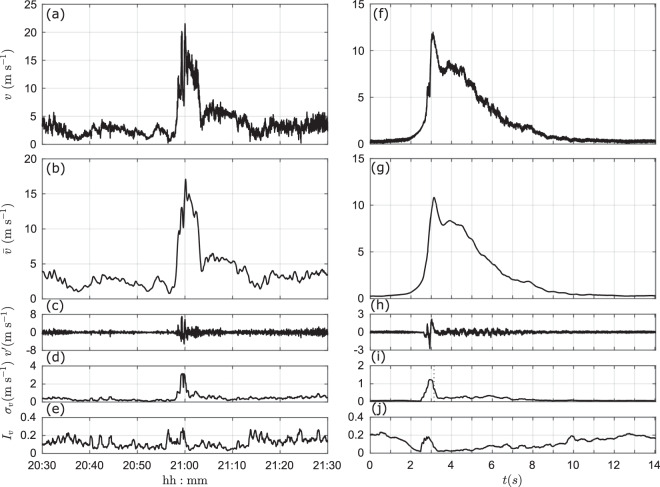
(**a**–**e**) Decomposed wind velocity of the full-scale downburst event occurred in Genoa on 30 September 2012 (fs); (**f**–**j**) Decomposed wind velocity of the WindEEE Dome DB8.9 case at the position (*r*/*r*_max_ = 1.4, *z*/*z*_max_ = 1.0) (exp). *v* is the 10-Hz (fs) and 2500-Hz (exp) instantaneous velocity; $\bar{v}$ is the 30-s (fs) and 0.1-s (exp) slowly-varying mean wind velocity; $v{\prime} =v-\bar{v}$ is the residual turbulent fluctuation; *σ*_*v*_ is the slowly-varying standard deviation of *v*′; ${I}_{v}={\sigma }_{v}/\bar{v}$ is the slowly-varying turbulence intensity. Figure from Romanic *et al*.^29^.

The effectiveness and reliability of the experimental procedures concerning the physical simulations of non-stationary winds carried out over the last 10 years in the WindEEE Dome has been validated by the large number of peer-reviewed publications on downburst and tornado winds (e.g.^28,29,33,35,49–52,61,63^). Comparisons between full-scale and experimentally generated downburst winds at the WindEEE Dome are present in the following studies^28,29,35,62^. For example, Fig. 6 is extracted from the study of Romanic *et al*.^29^, where the authors analyzed a downburst event that occurred in Genoa on 30 September 2012. This full-scale event is here compared against the DB8.9 downdraft measured at the position (*r*/*r*_max_ = 1.4, *z*/*z*_max_ = 1.0). The decomposed wind velocity shows overall good agreement between real and model cases, both in terms of mean wind velocity and turbulence. Further comparisons against several other full-scale events, as well as a scaling methodology that can be used to properly compare WindEEE Dome experimental results and actual downburst measurements is reported in Romanic *et al*.^29^.

## Usage Notes

The database of measurements published at the online repository PANGAEA contains two files—Velocity_sdb8_9.txt and Velocity_sdb16_4.txt—that correspond to two different jet velocities tested in the experiments, namely, *W*_jet_ = 8.9 and 16.4 m s^−1^. The format of the files is ASCII text. Each of the two sub-datasets contains 10-s time series of the three components of wind speed at different heights and radial positions. Data acquisition frequency was *f*_*s*_ = 2500 Hz. Each column refers to a specific velocity component at a given height and is named according to the following convention: VCOMP_zXXcm[m/s]; where VCOMP is one of the three velocity components (*U*, *V*, *W*). The velocity triplet represents radial, transversal and vertical components, respectively, whose positive directions are reported in Figs. 2 and 3, and XX is the height of the Cobra probe on the mast given in centimeters. The last two columns refer respectively to the repetition number # and radial position *r/D* of the measurement.

While possible use cases of the dataset are described in ‘Background & Summary’ section, the following paragraph discusses some of the limitations related to the physical simulations of downbursts in general as well as about WindEEE Dome. The first important limitation in any physical simulation is the dynamic scaling as expressed by the Reynolds number. *Re* at WindEEE is two or three orders of magnitude lower compared to the full-scale downburst phenomena. While Reynolds number independency about a certain critical *Re* has not been specifically demonstrated for downburst simulations, it has been demonstrated for tornado simulations at WindEEE at similar geometric scales^51^. The second important limitation is related to boundary conditions, which again is common for all physical simulators. WindEEE Dome is the largest simulator of its kind, therefore the influence of the enclosure boundary conditions is considered to be acceptable. Thirdly there are limitations related to other minor physical/mechanical factors at the WindEEE Dome such as: (i) The outer edge of the bell mouth and its louvres create flow disturbances. However, an ongoing numerical study has demonstrated that the outflow is not affected by these mechanical components of the WindEEE Dome simulator. (ii) The opening/closing mechanism of the louvres at the bell mouth is not symmetric in respect to its center. When they open, the rotate 90° onto the vertical plane from 0° (horizontal/closed position) to 90° (opened position). While this introduces some asymmetries, as mentioned in the ‘Velocity measurements setup’ sub-section, measurements were taken along the azimuth line identified by *α* = 90° (see Fig. 2a), and therefore they are negligibly affected by the opening/closing asymmetry. (iii) The testing chamber of the WindEEE Dome presents an asymmetric vertical structure given by the presence of the 60-fan (4 × 15) matrix on one of its peripheral walls while there are only 8 fans at the base of each of the other 5 walls. However, during downburst simulations all the louvres on the 60-fan wall, with the exception of the ones corresponding to 8 fans at the base, are kept closed. This is believed to produce the least non-symmetry in the flow.

Data can be further reused under Creative Commons license CC0 for metadata and CC-BY for data to widen the investigation.

## Data Availability

The experimental measurements were processed using a MATLAB^®^ library internally built by the Cobra probe manufacturer Turbulent Flow Instrumentation Pty. Inc. All custom scripts that were used to synchronize wind measurements across different experiment repetitions (“1_alignSignals.m”), to save the data in proper format (“2_saveData.m”), and to generate the database (“3_datasetWriting.m”) have been made available^36^. All scripts were created in the commercial software MATLAB^®^ (version R2017b).
